# Feasibility of a Study Within a Trial to evaluate a decision support intervention for families deciding about research on behalf of adults lacking capacity to consent (CONSULT SWAT)

**DOI:** 10.1186/s13063-025-09021-3

**Published:** 2025-08-27

**Authors:** Victoria Shepherd, Kim Smallman, Fiona Wood, Katie Gillies, Adam Martin, Maria Moore, Stacy Todd, Kerenza Hood

**Affiliations:** 1 Centre for Trials Research, Cardiff University, Wales, UK; 2 PRIME Centre Wales, Wales, UK; 3 Division of Population Medicine, Cardiff University, Wales, UK; 4 Aberdeen Centre for Evaluation (ACE), University of Aberdeen, Aberdeen, UK; 5 Academic Unit of Health Economics, University of Leeds, Leeds, UK; 6 Liverpool School of Tropical Medicine, Liverpool, UK; 7 Tropical and Infectious Disease Unit, NHS University Hospitals of Liverpool, Liverpool, UK

**Keywords:** Informed consent, Clinical trial, Proxy decision making, Study within A Trial, SWAT, Embedded randomised controlled trial

## Abstract

**Background:**

Trials involving adults who lack capacity to consent can be challenging, partly due to the involvement of ‘proxy’ decision-makers. This is usually a family member, who advises the researchers about the person’s wishes. Families can find decision making difficult and some experience a decisional burden. Following the development of a decision aid for family members making trial participation decisions, we are conducting a mixed-methods randomised Study Within a Trial (SWAT) to evaluate its (cost-)effectiveness. This paper reports the feasibility stage conducted in one host study to inform delivery of the main SWAT.

**Methods:**

Family members approached to act as a consultee for the host study were randomised 1:1 to receive the decision aid booklet alongside standard study information (intervention), or standard information plus a blank notebook (control), and asked to complete the CONCORD scale (Combined Scale for Proxy Informed Consent Decisions) questions about their experience and take part in a semi-structured interview. Acceptability of the SWAT was assessed through exploring recruitment rates and data completeness, and qualitatively through interviews with family members and research staff. Interviews were analysed using a rapid qualitative approach.

**Results:**

In total, 92 family members were randomised to the SWAT and 16 completed the CONCORD questionnaire. Interviews were conducted with consultees (*n* = 4), and host study staff (*n* = 3) who also provided resource use data. Differences in time staff spent with consultees were small.

Key themes included (1) setting up the SWAT and balancing priorities with the host study, (2) differences when recruiting consultees to a SWAT, (3) feasibility and acceptability of the SWAT, (4) challenges of measuring decision quality and (5) views and experiences of the decision support intervention.

**Conclusion:**

The CONSULT SWAT is feasible, but changes to study processes are needed in advance of the main SWAT. The findings suggest that attempting to seamlessly integrate the SWAT into the host study may have inadvertently led to it becoming ‘invisible’ to consultees. The small number of trials involving participants lacking capacity limits opportunities for developing the evidence-base. Recruitment of host trials continues, with a focus on evaluating the intervention in a broad range of populations and settings.

**Trial registration:**

The SWAT is registered as SWAT #159 with the Northern Ireland Hub for Trials Methodology Research SWAT repository (registered 09.08.2020).

**Supplementary Information:**

The online version contains supplementary material available at 10.1186/s13063-025-09021-3.

## Background

Some potential trial participants lack capacity to make an informed decision about taking part in trials due to conditions which impair their ability to understand, retain, weigh up or communicate their choices about joining a research study. Conditions which can result in a lack of capacity can include dementia, mental health conditions, learning disabilities, palliative care or other acute or emergency conditions. Where an adult is deemed to lack capacity to consent to a trial, alternative consent arrangements are required. In the UK, the legal frameworks permit the recruitment of adults lacking capacity based on the involvement of an alternative decision maker, provided that appropriate ethical approval has been obtained [1].

The clinical trial regulations governing investigational medicinal products (CTIMPs) in the UK [2] differ from the legal frameworks governing trials not classed as CTIMPs which are covered by the mental capacity legislation applicable in that jurisdiction [3–5]. Whilst all these legal frameworks require the involvement of a person with a relationship to the participant prior to their inclusion in non-emergency research, their role in the decision and the terminology used differs [6]. Under the clinical trials regulations, researchers must identify someone who cares for the person to act as a legal representative to provide consent on the person’s behalf [2]. Ordinarily, this would be a family member or close friend who is asked to act as a personal legal representative. For non-CTIMPs in England and Wales, under the Mental Capacity Act, a family member or close friend acts as a personal consultee and provides advice to the research team, who are then responsible for making a decision about participation [3]. In both cases, consent from the legal representative or the advice from the consultee must be based on what the person themselves would have wished if they had capacity to decide (their ‘presumed will’). There are also arrangements for situations where there is not a family member or close friend willing or able to act as a personal legal representative or personal consultee, in which case a professional (e.g. a doctor or another member of the care team) can act as a professional legal representative or nominated consultee, provided they are independent of the research project.

Families acting as a legal representative or consultee often find making decisions about what the person would have wanted challenging [7–9], partly because discussions about research preferences between family members are uncommon. Families have described the decisional and emotional burden of acting as a legal representative/consultee [7], which can lead to them being more likely to decline participation than patients do themselves [10]. It also raises concerns that the decisions they make about trial participation may not reflect the person’s wishes and preferences. These issues mean that adults lacking capacity are often excluded from trials, including in conditions where there is a high prevalence of cognitive impairment, leading to findings that are not relevant to the actual clinical population [11]. Those studies that do include them often recruit only small numbers who lack capacity, resulting in them being underpowered or abandoned.

There have been calls for better inclusion in research of adults lacking capacity to consent who are recognised as being an under-served group in research by organisations such as the National Institute for Health and Care Research (NIHR) [12], Nuffield Council on Bioethics [13], and the World Health Organisation [14]. Families have called for more support when making decisions about research on behalf of someone who lacks capacity to consent [7] and researchers have identified the need for interventions to support them to conduct research involving this population, which they find challenging [15].

**Fig. 1:**
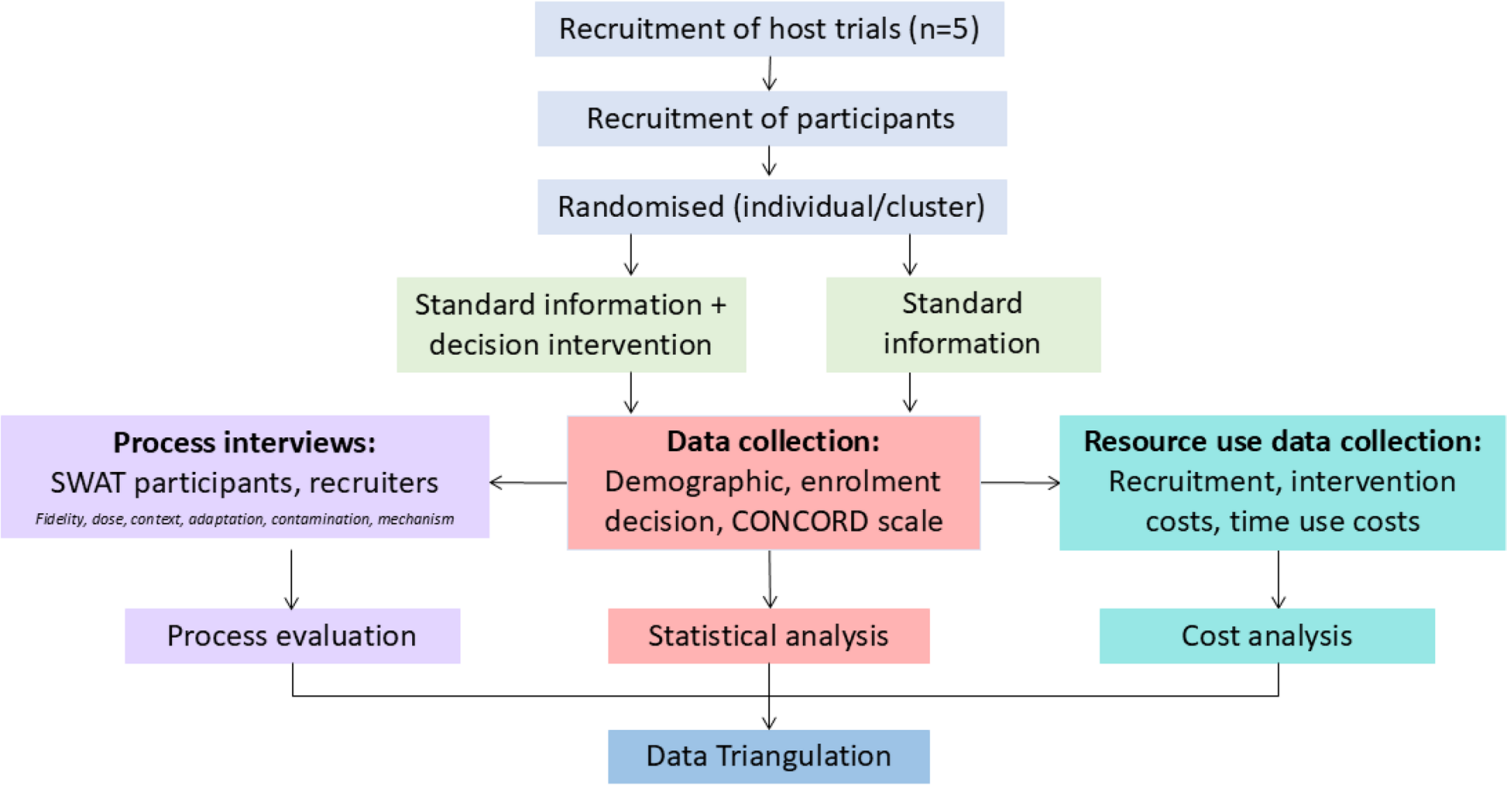
CONSULT SWAT trial design

To date, recruitment interventions have focused on participants who are able to provide their own consent (such as [16–18]), or parents of children in paediatric studies. As part of the larger CONSULT research programme which explores the ethical, legal and methodological issues around research involving adults with impaired capacity to consent [19], a novel decision support intervention was developed for families acting as a legal representative/consultee [20]. The intervention consists of a decision aid (DA) that is intended to support family members and help them make an informed decision about participation, alongside brief training for researchers approaching legal representatives/consultees. The intervention is being evaluated using ‘Study Within a Trial’ (SWAT) methodology [21]. SWATs are self-contained research studies that are embedded within a host trial with the aim of evaluating alternative ways of delivering or organising a particular trial process [22]. They can be conducted across multiple host trials, either at the same time or sequentially. The CONSULT SWAT is exploring the effectiveness of the DA in up to five host studies, with an embedded process and economic evaluation [21] (see Fig. 1).

As evaluations are often undermined by problems of acceptability, compliance, intervention delivery, recruitment and retention, a feasibility study can enable these issues to be explored and addressed prior to any larger-scale evaluation. Given that the novel intervention in the CONSULT SWAT is aimed at representatives/consultees rather than trial participants themselves, it is anticipated that the SWAT will encounter issues not experienced in previous SWATs [23] which therefore merit additional exploration. This paper reports the findings from the feasibility stage of the CONSULT SWAT which was conducted in one host study in order to inform the ongoing delivery of the main SWAT and to provide insights for future SWATs. Provided no changes are required that materially changes the SWAT components, data from the feasibility stage will be subsequently included in the meta-analysis.

## Methods

The study design and methods for the CONSULT SWAT, including the feasibility stage, are described in the study protocol which has been published [21]. Briefly, the CONSULT SWAT is a two-arm, parallel group, embedded randomised-controlled trial, with an allocation ratio of 1:1 to investigate the effect of a decision support intervention compared with standard study information on decision-making by consultees and legal representatives of adults lacking capacity to consent. The SWAT is registered as SWAT #159 with the Northern Ireland Hub for Trials Methodology Research SWAT repository [24].

The feasibility stage was designed to inform the main SWAT and help to determine whether any changes to SWAT processes are required, rather than being assessed against formal ‘stop/go’ criteria [21].

The feasibility stage aimed to:

1. Assess the acceptability of the intervention and SWAT procedures and identify any unintended consequences
2. Estimate the likely rates of recruitment and retention of participants for the SWAT
3. Assess completion of the outcome measures and resource use information
4. Explore the likely cost of delivering the intervention in a trial setting

The central SWAT team (led by author VS) developed the SWAT protocol and processes and co-ordinate the SWAT across host studies. The SWAT is delivered in each host study by the host study team who set up the SWAT at sites, recruited participants to the SWAT and the host study, provided the decision support booklet and collected data including the CONCORD questionnaire. Qualitative interviews with consultees and members of the host study team were conducted by the central SWAT team (author KS).

Ethical approval for the overarching SWAT has been obtained from Leeds West Research Ethics Committee (ref. 22/YH/0121), with host studies obtaining amendments to their approvals for embedding the SWAT. The findings are reported in accordance with SWAT reporting guidelines (Trial Forge Guidance 4) [25] and for feasibility studies [26].

### Participants

#### Host study

Invitations to act as a host study for the feasibility stage of the SWAT were distributed via research networks (e.g. UKCRC Clinical Trials Unit network), social media (e.g. Twitter/X), and via trials methodology research networks (e.g. MRC-NIHR Trials Methodology Network) and the CONSULT website. Host study teams were offered practical support to help embed the SWAT in their study, including template text to add into the study protocol, and funding of up to £5000 to cover SWAT-related activity such as obtaining any additional approvals.

Studies were eligible if the host study team anticipated that a ‘reasonable proportion’ of potential participants would lack capacity to consent (to be determined through discussion between the CONSULT and host study teams), and personal consultees or legal representatives were involved in decisions about participation on their behalf. Studies were excluded if the participation decision needed to be made urgently or within a short timeframe for the purposes of the study (i.e. emergency research including studies involving recruitment without prior consent (e.g. ‘deferred’ consent)), or where only professionals acting as nominated consultees or professional legal representatives were involved in participation decisions.

#### Participant eligibility criteria

Participants for the SWAT were recruited through the host study in accordance with their processes for recruiting participants who lack capacity to consent, who were also responsible for assessing participant eligibility for the SWAT.

Participant inclusion criteria:

- Family member or friend approached to act as a personal consultee or legal representative on behalf of a participant eligible for the host trial
- Able to read and understand English sufficiently well to comprehend study information and decision aid booklet
- Able to provide consent to participate in the study

Participant exclusion criteria:

- Professional approached to act as a nominated consultee or professional legal representative
- Has previously participated in the CONSULT SWAT

### Intervention

The development of the decision aid has been previously reported [20]. Briefly, it is a 12-page A5 colour booklet (‘Making decisions about research for others’) which is provided to family members in addition to standard information about the host study. It is intended to be used by the family member at the time they are making a decision about whether the person they represent should participate or not. It contains an explanation about why they are being approached, why adults lacking capacity are included in research, and a six-step guide to making a decision. It also includes a values clarification exercise to help them to understand what the advantages and disadvantages might be, and to consider how the person they represent would view them and come to a decision about participating or not. The control is standard study information, alongside a blank notebook that matched the size and weight of the decision aid booklet.

As studies are increasingly using remote methods for recruiting participants, including contacting families acting as legal representatives/consultees, an electronic version of the DA was developed by a specialist graphic design company. This is an interactive document (PDF) that can be attached to an email if that is the method used by the host study to approach families.

Training was developed by the central SWAT team as bitesize modules for host study staff which were audio-recorded PowerPoint presentations which covered the background to the intervention and SWAT, delivery of the intervention, and consent and data collection processes. A site manual was developed to accompany it, with suggested phrases for members of the host study team to use when approaching consultees and template text for emails to be sent to those who preferred that contact route.

### Outcomes

Acceptability of the SWAT was assessed through recruitment rates as measured through recruitment logs (recording the number of family members identified and approached to act as a consultee who were randomised to the SWAT) and return of the CONCORD scale questionnaire, evaluating completeness of CONCORD data, and qualitatively through interviews with family members and research staff (recruiters and members of the trial co-ordination team).

The primary outcome measure for the main SWAT is a newly developed scale to assess the quality of ‘proxy consent’ decisions made about trial participation by family members on behalf of someone who lacks the capacity to consent for themselves (CONCORD) [27], with higher scores indicating higher decision quality. It will be concurrently validated during the SWAT. Secondary exploratory outcomes include selected CONCORD subscales of values clarity and preparedness, and the proportion of consultees and legal representatives who provide agreement to participate on the person’s behalf and subsequent retention in the host trials.

### Sample size

There are generally no formal a priori sample size calculations for SWATs [22], as they are usually undertaken on the basis of the maximum number of recruiters and participants possible per host study. The estimated sample size is based on similar feasibility studies of novel complex interventions, aiming to conduct the feasibility stage in one host study with 20 family members and up to 15 research staff (recruiters and members of the trial co-ordination team) depending on the size of the host study team.

In order to inform the acceptability of the SWAT to detect any biases from differential recruitment, sites were asked to complete a screening log of all potential participants who were eligible for the SWAT but did not consent or were not approached. Sites were also asked to maintain a recruitment log of all participants enrolled in the SWAT, and their allocated PID.

### Randomisation

Participants were randomised 1:1 to receive the DA booklet alongside the standard information provided to consultees/legal representatives by the host study, or standard information with a blank notebook. The allocation sequence was generated centrally by the Centre for Trials Research (CTR), who co-ordinated the SWAT, using an online randomisation tool (www.randomizer.org). Pre-prepared packs were provided to the site teams who selected the next sequentially numbered pack and provided it to the family member when consulting them about the host study. The allocation was concealed from the member of the site team who was approaching the family member prior to providing the family member with the pack.

Where family members were contacted by the site team by email rather than in person, a pre-prepared allocation list was used and participants were randomised to receive the interactive PDF version of the DA alongside the standard information, or standard information alone. These documents were attached to a standard email sent by the recruiting member of the host study team. It was not possible to conceal the allocation from the site team members in these circumstances.

### Data collection

After receiving the intervention (or control), family members were asked to complete a questionnaire which included questions about their relationship to the person they represented and the length of time they had known them, their use of the information they had received (standard information with or without the DA), whether they had agreed to participation on the person’s behalf or declined, and the CONCORD scale. A numerical linking ID was used to collect pseudonymised data on the recruitment and retention of the host trial participant. The questionnaire was provided in hard copy where possible, or via an individualised online link (Qualtrics) in an email depending on how the site team were communicating with the family member.

Family members could provide their contact details via the questionnaire if they were willing to be contacted about taking part in an interview with the central CONSULT SWAT team. Members of the site team were also invited to take part in a semi-structured interview. Interviews were conducted remotely (Teams) by an experienced qualitative researcher (KS) who was not involved in the development of the intervention. Verbal consent was obtained prior to commencing the interview. A topic guide (see Appendix 1 for version for interviews with family members) was developed by the research team and used to help structure the interviews, which were audio-recorded, transcribed verbatim by an external transcription service, and pseudonymised prior to analysis. See the CONSULT SWAT protocol for more details [21].

Resource use data were collected using a questionnaire developed for the SWAT and completed by the member of the site team who was approaching the family member. This elicited information on the role and grade of the person contacting the family member, how this was done (e.g. face to face consultation or by phone), and the time required for the consultation and discussion.

### Data analysis

CONCORD scores were transformed to a 0–100 scale, where higher scores indicate higher decision quality. Descriptive statistics were used to assess levels of data completeness and scoring of the CONCORD scale and individual items within the scale. This was to help inform sample size expectations for the main SWAT and explore the likely minimal methodologically important difference (MMID). Resource use data were also reported descriptively.

Qualitative data were analysed using a dual approach: rapid qualitative analysis which enabled the development of timely and actionable findings to inform changes in practice or process whilst data collection was still ongoing [28], and thematic analysis [29] to provide an in-depth understanding of participants’ views about the acceptability of the SWAT, intervention and outcomes.

For the rapid analysis, RREAL sheets were used as a tool to organise the data, facilitate the identification of any gaps during data collection, support collaborative interpretation and sense-making of findings and help synthesise the findings to enable them to be shared with the CONSULT SWAT research team in real time [30]. The RREAL sheet templates were developed by two members of the CONSULT SWAT research team (VS and KS), with different versions for interviews with consultees (Appendix 2) and staff. They were completed by the researcher conducting the interview (KS), and the key themes discussed. These were then used to provide feedback to the site team and to make amendments to study processes to address any issues that were identified.

For the thematic analysis, transcripts were iteratively coded by one researcher (KS) who was blinded to the allocation and organised into themes which were developed and finalised through discussion with a second researcher (VS). The findings from the thematic analysis were discussed with the wider research team, alongside the rapid analysis findings, to inform the ongoing SWAT.

## Results

### Recruitment of the host study

As the invitation to act as a host study was distributed via a range of routes including social media and the CONSULT website, the number of potentially eligible studies and the reasons for studies deciding whether to participate or not are unknown.

Recruitment of the host study was impacted by the relatively small number of eligible studies and coincided with the NIHR’s Reset programme to get the research system back ‘on track’ following COVID which led to enhanced scrutiny of recruitment rates and closure of studies not reaching particular milestones [31]. This led to potentially eligible studies being concerned about introducing an additional SWAT, and some were prematurely closed.

Where studies responded to the invitation to participate (*n* = 11), those that declined the offer to embed the SWAT (*n* = 4) cited concerns around the potential impact on the host study (e.g. additional workload for the team, participant burden, being monitored by the funder due to recruitment issues), or a perception that the decision support intervention was not needed (e.g. view expressed by public involvement contributors), or that the trial context was too sensitive (e.g. end of life care) or a combination of these.

Of the studies who agreed to participate (*n* = 7), most were not in a position to be part of the SWAT feasibility (*n* = 6) due to timing as they were either still in the set up phase (e.g. had an initial intervention development phase), were waiting for an internal feasibility stage to be completed, or had yet to secure funding but were including the SWAT in their funding application.

This meant that for pragmatic reasons the study selected to host the SWAT for the feasibility stage was an observational study, rather than a clinical trial, although it involved the collection of clinical samples which would require consideration by consultees when deciding about participation on the person’s behalf. The host study (anonymised to avoid identifying participants in the SWAT due to the small numbers involved) was exploring antimicrobial resistance in older people and recruited older people in two types of care settings (care homes and hospital) in one region of England. The study involved the collection of clinical samples (swabs from the environment, stool samples or rectal swabs from residents/patients, swabs from the hands of staff) and data from hospital and GP records. A data transfer agreement was put in place between the sponsor of CONSULT SWAT and the organisation responsible for co-ordinating the host study.

All patients or residents in the study sampling location during the sampling period were eligible for the host study. Due to the nature of the study population, it was anticipated that a significant number of people would lack capacity to consent to the study; therefore, patients/residents with and without capacity were recruited. In accordance with the Mental Capacity Act [3], where a patient/resident was assessed as lacking capacity to consent, a family member (or close friend) was identified and approached to act as a personal consultee to provide advice about whether the person lacking capacity should take part in the study. At this point, the family member (or close friend) became eligible for the SWAT. As the decision aid is intended for use at the point at which a family member makes a decision as a consultee, the SWAT used a pre-consent randomisation design, in which they were allocated to the intervention or control group prior to agreeing to participate in the SWAT (indicated by return of the questionnaire which constituted consent to participate in the SWAT).

When the host study team approached a family member to act as a consultee, the host study was introduced first, with an explanation about why they are being approached and what the study is about. They would also inform the family member that as well as being approached about their relative taking part in the host study, they were being invited to take part in a separate study to gather their views about the information they were being given when making a decision about the host study.

The family member was then provided with the information about the host study (Consultee Information Sheet) and brief information about the CONSULT SWAT which including information about who their data will be shared with and how it will be used and stored. Depending on their allocation, they were also provided with the decision support booklet (intervention) or blank booklet (control) in a sealed envelope. Different arrangements applied for those approached by phone or email as outlined above.

Prior to opening the envelope, the member of the host study team advised the family member acting as consultee that they were being provided with an additional item, for example “Before you make a decision there is something to help you think through the study information”. They were then encouraged to use the booklet they had been provided with as they made a decision about the host study, for example “You can use this to think about the study and what is involved and to write down any questions you have”. If they were allocated to the decision support booklet, the member of the host study team was instructed to signpost them to the different sections of the booklet and encourage them to complete the sections of the booklet as they went through it. This includes the values clarification exercise in Part Four where they are encouraged to reflect on what the person’s views would be about the benefits or disadvantages of taking part. For example, for this host study, it might involve their views about providing clinical samples or data or contributing to helping other patients/residents in the future. It was also suggested that they retain the completed booklet as they may wish to return to it in future.

The member of the host study team then answered any questions the consultee had about the host study or the SWAT. If the consultee agreed that the patient/resident would want to participate in the host study, they completed a Consultee Declaration Form. If they were willing to take part in the SWAT, they were asked to complete the questionnaire that was attached to the brief information about the SWAT. Return of the questionnaire constituted consent to participate in the SWAT.

### Feasibility of recruitment to the SWAT

#### Recruitment and retention data

The host study was open to recruitment between March 2023 and June 2024 and in total recruited 161 participants, of whom 54 lacked capacity to consent. Recruitment to the SWAT started in September 2023 due to the host study team not yet having staff in post and then wanting to build confidence around the approach and consent process (for both studies) prior to introducing the SWAT.

**Fig. 2:**
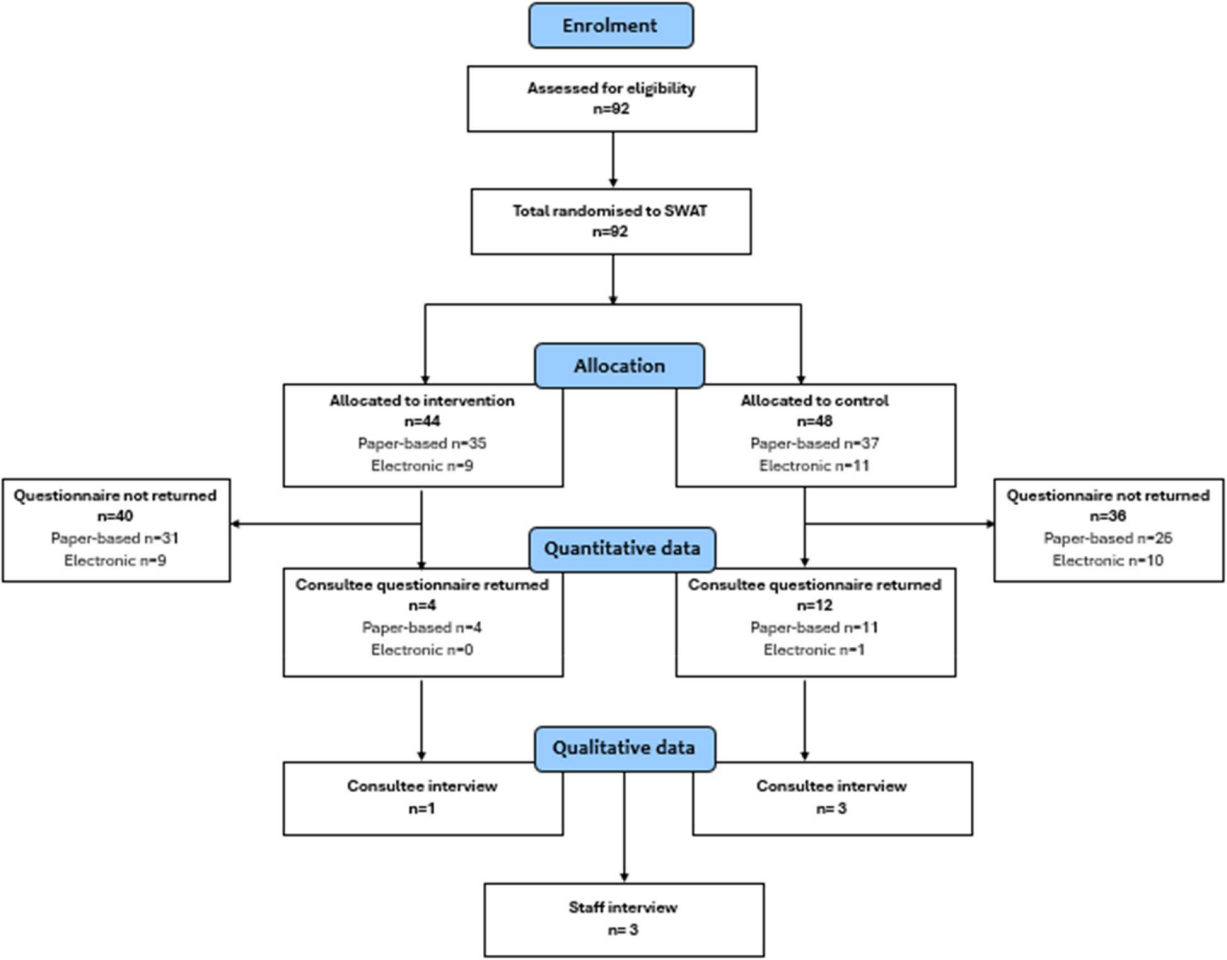
CONSORT flow diagram

Between September 2023 and June 2024, 92 family members were randomised to the SWAT (see Fig. 2 CONSORT flow diagram), having been identified as potential consultees for patients/residents who were eligible for the host study but lacked capacity to consent. Of these, 16 consented to participate in the SWAT (defined as having completed and returned the CONCORD questionnaire which constituted consent to participate), of whom six agreed to participate in an interview.

Interviews were conducted with four consultees (one subsequently declined, and one did not respond when contacted). Three interviews were also conducted with the two members of the host study research team, one staff member was interviewed at two different time points to explore changes in their experience of the SWAT during the earlier and later stages of implementation.

Participant characteristics are shown in Table 1.

**Table 1:** Participant characteristics

	Participants ***n* = *16 (%)***
Relationship to the person being consulted about	
Child	9 (57%)
Sibling	3 (19%)
Spouse/partner	2 (12%)
Cousin	1 (6%)
Unknown	1 (6%)
Length of relationship with the person they represent	
Length of time (mean years)	53 (range 18–76)
Mode of receiving CONSULT SWAT study information	
In person	15 (94%)
Unknown	1 (6%)
Proportion of all study information materials read	
Had read all the information	13 (82%)
Had read some of the information	1 (6%)
Unknown	2 (12%)
Mode of further contact with host study research team^	
In person	12 (75%)
By phone	6 (37%)
Unknown	1 (6%)
Length of time taken to consider host study	
Length of time (mean minutes)	39 (range 0–180)
Other (e.g. text response)	*n* = 5 (e.g. “after reading through”)
Unknown	*n* = 1
Outcome of the consultee decision	
Agreed to person taking part	16 (100%)
Declined for the person to take part	0

^ Participants could select more than one mode of contact

Of the 92 family members who were randomised to the SWAT, 41 agreed to act as consultee and completed a Consultee Declaration Form to confirm that they agreed to the patient/resident participating in the host study. Of the remaining 51 family members, the exact number who either did not respond, declined to act as consultee, or declined the study on the person’s behalf (in which case the Consultee Declaration Form was not completed) are difficult to ascertain, but none agreed to participate in the SWAT.

Recruitment and retention logs completed by the host research team showed that of the 41 consultees who agreed to the patient/resident’s participation in the host study, no consultees in the intervention arm withdrew the participant from the host study. One consultee from the control arm did withdraw a participant.

#### CONCORD questionnaire completion

All 16 consultees who completed the CONCORD scale (the primary outcome measure for the main SWAT) provided complete responses across all 28 questions in the scale. Most consultees (*n* = 13) used a single response category for all questions (e.g. either ‘strongly agree’ or ‘agree’) or two categories, meaning there was little variation across individuals’ responses. CONCORD scale data is reported below as an exploratory outcome for this feasibility stage of the SWAT.

#### Resource use

Resource use data was available for 29 of the 92 consultees who were randomised to the SWAT. The time taken to provide information to family members acting as consultees and seeking their advice was similar between groups, with a median time of 5 min (IQR 5–10) in both the intervention group (*n* = 15) and in the control group (*n* = 14), with no difference in the grade of staff involved or their professional background.

#### Intervention costs

The main cost of the intervention is for colour printing the hard copy decision aid booklet or notebook (£0.75 per copy) and packing and postage to the study site (£3.99 and £4.19 respectively for 120 participant packs) which was arranged by the team co-ordinating the SWAT and covered by the CONSULT study budget. This equates to a cost of £0.82 per potential SWAT participant for the intervention materials. Costs were also provided to the host study (£5,000) to support SWAT-related activity such as preparing and submitting the substantial amendment. It is not known if this corresponds with the actual costs incurred by the host study as a breakdown of costs was not required from host studies in order to reduce the administrative burden.

### Exploratory findings

#### CONCORD scale

There was an overall mean CONCORD score of 92.4 (range 75*–*100) (see Table 2), with higher scores reported in the control group (mean score 94.5) compared with the intervention group (mean score 87.5).

**Table 2:** Summary of CONCORD scores

	Intervention			Control			Overall		
	No. of consultees	Mean	Range	No. of consultees	Mean	Range	No. of consultees	Mean	Range
CONCORD score	4	87.5	75–100	12	94.5	75–100	16	92.4	75–100

### Qualitative findings

Participants reported their experiences of being involved in conducting the SWAT, the recruitment process for the SWAT and host study, and of participation in the SWAT. A number of key themes were identified, including (1) setting up the SWAT and balancing priorities with the host study, (2) differences when recruiting consultees to a SWAT, (3) feasibility and acceptability of the SWAT, (4) challenges of measuring decision quality and (5) views and experiences of the decision support intervention. These are summarised in Table 3.

**Table 3:** Summary of key qualitative themes

Themes and sub-themes	Description of themes and sub-themes	Illustrative quotes
Setting up the SWAT and balancing priorities with the host study	The host study team needed to balance the competing demands of setting up and delivering both the SWAT and the host study	“…*there was a time pressure on us for [host study]. We had to recruit our first participant within a certain time limit, and I was keen to get that done…I didn’t want to slow that process down by introducing a second*.” [SI1, member of host study team]
Differences when recruiting consultees to a SWAT	There were key differences between recruiting to the SWAT and the host study, and to other types of SWATs	-
- Approaching consultees	The process for approaching consultees aligned with, but differed from, the process for recruiting participants for the host study	“*It is tricky. You know it’s quite complicated because I’m aware of the fact that … your participants … are not our participants*.” [SI1, member of host study team]
- Minimising the burden for consultees	The host study team attempted to minimise the burden for consultees and avoid overloading them with information about the two studies	“*If you go through two studies, people are just going to be completely bamboozled, you know they’re not even thinking about one study, they’re thinking about how to get their elderly relative home because that’s the core of people that we’re dealing with*.” [SI1, member of host study team]
- Barriers to recruitment to the host study and SWAT	Studies involving consultees can be more challenging, which presents particular barriers when recruiting to the SWAT	“*Sometimes, once they’ve signed the consent, we have nothing else to do with them. We don’t speak to the next of kin [again]. Everything is done and dusted then isn’t it, so we don’t always see them again after they’ve signed the consent form. Depending on what day it is or how much longer we’ve got in the area that we are in*.” [SI2, member of host study team]
- Facilitators to recruitment to the host study and SWAT	The host study team identified a number of strategies to aid recruitment to both the host study and the SWAT	“*They do a family meeting every month, so when we were coming in, most of them were already aware that we were coming, because they’d attended the family meeting and we’d been spoken about in that meeting—when we were coming in, what we were going to do, what we were looking to do, the reasons why we were doing it. That was a really, really helpful tool for us because we were already introduced*.” [SI2_I2, member of host study team]
Feasibility and acceptability of the SWAT	Consultees appeared to find the SWAT acceptable, but were not always able to distinguish it from the host study	“*It felt like it was part of the consent process really. You know, gathering of information and consent*.” [1056, consultee, control group]
Challenges of measuring decision quality	Consultees sometimes mistook completing the CONCORD scale with being part of the consent process, which affected the data collected	“*It was all together, and I wasn’t aware which bit was what*.” [1015, consultee, control group]
Views and experiences of the decision support intervention	Consultees described their experiences of making research decisions and their views about the decision support booklet	-
- Making decisions about research	Consultees thought that taking part in research was important, but even when they were very experienced in making decisions on the person’s behalf, they still expressed uncertainty about whether they were doing ‘the right thing’	“*I know that this is something that [sister’s name] would, if she’d been approached when she was in her sound mind, certainly would have agreed about. So, I feel confident to say, yes, go ahead with this study, because I really believe that it’s what she would have done. We’ve made so many decisions on her behalf, that you would have thought we would have been quite comfortable, but each decision we have to make, it’s still, have we done the right thing*?”” [1028, consultee, intervention group]
- Decision support booklet	Consultees often struggled to recall the decision support booklet, but overall had a tendency towards positive views	“*I think we argued the toss about it, you know. Should we or shouldn’t we, you know. We know a little bit about what is involved, we did ask questions at the time about it and were reassured. It must have been okay, because we went ahead. I can’t remember thinking any sort of adverse things about it*.” [1028, consultee, intervention group]
- Delivery of the decision support intervention	The host study team generally provided consultees with the decision support booklet without much interaction or engagement with it, however they did describe a change it had on their attitude and practice	“*We’re presenting it together with our study information. So, probably in practice we’re not going through it perhaps in the same way that was originally intended*.” [SI, member of host study team] “*The initial reaction [from consultees] was ‘oh, that’s a good thing to have’. Because [being a] consultee is difficult, and no-one prepares you for making that decision on behalf of somebody else. It’s certainly made me think about it*.” [SI, member of host study team]

### Setting up the SWAT and balancing priorities with the host study

In this feasibility stage, host study team members described the process for adding the SWAT to the protocol of the host study and gaining ethical approval to be relatively straightforward. The study manager for the host study played a key role in embedding the SWAT including ensuring that the research staff delivering the host study were also trained to deliver the SWAT.

“It was simple to add into the protocol, and you’d already got REC approval and everything and that’s really helpful that you can present it as this is a study that you know is standalone.” [SI1, member of host study team].

Including the externally co-ordinated SWAT meant that there was a perceived need to balance the priorities of the host study in terms of their recruitment targets, particularly during a time of enhanced scrutiny by funders and research delivery networks. This meant that it was preferable to the host study team to open the host study and start recruiting their participants before including the SWAT in their processes.

“…there was a time pressure on us for [host study]. We had to recruit our first participant within a certain time limit, and I was keen to get that done…I didn’t want to slow that process down by introducing a second.” [SI1, member of host study team].

The set-up period involved a familiarisation process with the SWAT, which was seen as key to its successful implementation. It was facilitated by the strong relationships and good communication processes that were established between the host study and SWAT team. This included developing a mutual understanding about the respective study aims and the demands of study management.

“You know I’ve found it to be okay and I think both [SWAT researchers] are so responsive and that really helps that you’re not working within a void, you know you’re working with people who are around and listening and aware of things.” [SI1, member of host study team].

### Differences when recruiting consultees to a SWAT

Including an externally co-ordinated SWAT into a host study added another layer of processes and activities, particularly during the recruitment phase. Recruiting consultees as SWAT participants followed the identification of a potential participant for the host study, then determining that they lacked capacity to consent, and identifying and approaching a family member to act as a consultee—at which point they became eligible for the SWAT and were randomised.

#### Approaching consultees

There were some initial teething problems whilst the host study team familiarised themselves with the SWAT and developed an understanding about how recruitment would best work in practice. This led to an initial hesitancy about recruiting participants for the SWAT. However, the process of recruiting to the SWAT was refined over time as staff became more familiar with the SWAT, and their confidence in the approach they were using grew as recruitment for both studies progressed. This included refining the process for approaching consultees and presenting them with information about both the host study and the SWAT.

“I did approach, erm, one gentleman about it, and it was before we’d changed the way that we’re doing the approach now, and that’s when we kind of found that it didn’t really work you know in terms of people were a bit clunky.” [SI1, member of host study team].

“Now we’ve got our wording together, we know exactly what we are going to say when we see the patient’s next of kin, exactly how to word it and then we are handing the paperwork over. We know exactly what we are giving them, so it does make it that little bit easier now that we don’t have to sort of sit there in a panic and go ‘oh god, have I forgotten to give them something, do I need to give them this?’ So yeah, it is a lot easier now.” [SI2, member of host study team].

Members of the host study reported how the SWAT and host study overlapped in terms of some of the processes and delivery, and where they diverged. For example, if a potential participant in the host study lacked capacity to consent or might lose capacity during their time in the study, the host study team obtained the name and contact number of a family member. This meant that they were anticipating being contacted by the host study team at some point, which facilitated being approached about the SWAT.

“They’re aware that we’re going to do that anyway for our study because if somebody’s capacity changes or something changes in the future we might contact them again and just say are you still happy for your mum to take part in our study type of thing.” [SI1, member of host study team].

However, there were notable divergences between the respective recruitment processes due to the different roles that family members had in the host study, where they acted as consultee on behalf of the host study participant, compared with the SWAT in which they were a participant in their own right. This meant that there was limited ongoing contact with consultees, unlike participants in the host study.

“It is tricky. You know it’s quite complicated because I’m aware of the fact that … your participants … are not our participants.” [SI1, member of host study team]

#### Minimising the burden for consultees

There was some concern expressed by the study team about the burden of including information about the SWAT alongside the host study information when approaching consultees, particularly as these were family members of a person with often complex health and care needs. This was particularly the case when recruiting in hospital settings, where the person’s health issues may be more acute or there were complex discharge arrangements being made. The host study team were mindful about how to best present the SWAT information without negatively impacting delivery of information about the host study.

“If you go through two studies, people are just going to be completely bamboozled, you know they’re not even thinking about one study, they’re thinking about how to get their elderly relative home because that’s the core of people that we’re dealing with.” [SI1, member of host study team].

These concerns about not overburdening family members during a time when they were focusing on their relative’s care meant that research staff made an active decision about when to approach people about the host study or not, which they described as being based on their experience.

“Especially in the hospital, if they come in really poorly or there’s a lot going on, because they’ve got to sort, for example, a nursing home out for them, and everything’s taking so long. Sometimes though, you can find that the next of kins are a bit too stressed, some of them feel that they’ve got too much to deal with, to even think about anything else.” [SI2_I2, member of host study team].

“I have approached somebody when the whole family were round the bed and they were basically having a case conference about how they got mum home you know, and the social worker was there and you just thought, this is not the right moment.” [SI1, member of host study team].

#### Barriers to recruitment to the host study and SWAT

There were challenges associated with recruitment to the host study and SWAT in particular settings, including the staffing levels, medical record systems, and visiting times in that setting. This impacted on their ability to identify and contact consultees, as research staff did not have direct access to them and so relied on the care team or medical records for information. Accessing medical records and making contact with consultees was time consuming and labour intensive, and staff described instances where the information was incorrect, or it indicated that the person’s next of kin were not to be approached.

“Some have paper notes, some use the NHS system, others use their own personal system. We can’t always get access to that so sometimes we try to get that through the staff. Sometimes they are too busy so it can be a bit of a nightmare.” [SI2, member of host study team].

“With the care home, they don’t tend to come to visit as often because they’re there all the time. Some families you see on a very regular basis, every couple of days, or sometimes you can see them every day. But in a hospital, people might come and visit to begin with and then sort of not as often, depending on the length of time that person has been in hospital.” [SI2_2, member of host study team].

However, recruitment was often described as being more dependent on the circumstances and personal characteristics of those involved rather than the setting, and they adapted their approach as a result.

“I think I have got different ways of approaching patients and relatives, in different areas, and I think it’s just automatic for me. You can tell by the person. As you approach you can sort of see if that person is approachable and then you know which sort of role to take.” [SI2_I2, member of host study team].

One of the challenges of limited access to consultees in hospital settings meant that study documents were left for family members to complete and would go missing. Whereas in nursing homes where residents are more settled, documents could be left in a designated space for the family. Additionally, staff continuity within nursing home settings was seen as a benefit, compared with more acute settings.

“I think what makes it easier in the nursing home is they’ve all got their own personal space [and] you’ve also got maybe one or two reception staff.” [SI2_I2, member of host study team].

Additional complications occurred when there was more than one family member named who could potentially act as a consultee or where there was conflict between family members.

“We have had [situations] where the NHS record states that the patient does not wish for next of kin to be contacted for any purpose whatsoever. Or we’ve had patients that have dementia, they haven’t got the capacity but, on their system, it says daughter or son doesn’t exist, please do not contact. So obviously at that point we don’t contact the next of kin. We’ve also had an instance where the family members are both next of kins but neither of them get on, so it’s which one to contact, which can be a little bit difficult to try and work out.” [SI2, member of host study team].

There were other differences due to the different priorities of the host study and SWAT, and the different roles that family members had, such as family members who declined to act as a consultee when approached or declined participation in the host study and therefore would not necessarily be followed up about participation in the SWAT.

“We’re presenting it together with our study information. They see the two studies are sort of quite linked or meshed together and some of them say no to both studies straightaway.” SI1, member of host study team].

Even when contact was made with consultees, host study staff described how it could be difficult to keep in contact with them as they would not usually have ongoing contact as part of the host study.

“Sometimes, once they’ve signed the consent, we have nothing else to do with them. We don’t speak to the next of kin [again]. Everything is done and dusted then isn’t it, so we don’t always see them again after they’ve signed the consent form. Depending on what day it is or how much longer we’ve got in the area that we are in.” [SI2, member of host study team].

Compared with in-person approaches to consultees, the host study team reported very low uptake in recruitment via electronic approaches. It was especially difficult to follow up with consultees via this route as the team often did not know in ‘real time’ if consultees had completed the electronic questionnaire which was managed by the central SWAT team. This meant that they relied on the SWAT team to communicate whether this had happened or not.

#### Facilitators to recruitment to the host study and SWAT

Adding electronic prompts about the SWAT into the main host study database was seen as helpful in recruiting to the SWAT, as was members of the research team having contact and links within different settings prior to recruitment. For example, some nursing homes held regular meetings with families to inform them about the host study and this meant that families had prior knowledge about the proposed research study and that they may be approached about it.

“They do a family meeting every month, so when we were coming in, most of them were already aware that we were coming, because they’d attended the family meeting and we’d been spoken about in that meeting—when we were coming in, what we were going to do, what we were looking to do, the reasons why we were doing it. That was a really, really helpful tool for us because we were already introduced.” [SI2_I2, member of host study team].

As part of the host study, members of the research team conducted initial visits to each site and obtained environmental swabs prior to approaching participants, which also provided an early opportunity for research staff to raise awareness of recruitment and start building a relationship with family members. This were viewed by the team as being a way of building understanding of the setting and potential participants.

“When we go into a setting, we do the environmental swab first generally before approaching the patients, and that’s quite helpful because they get to see you walking around and they ask questions and you have a little chat with them, and that helps you to know whether you can approach that person or not.” [SI2_I2, member of host study team].

### Feasibility and acceptability of the SWAT

Consultees were very positive about their experiences with the consent process for the host study. They reported that the low-risk nature of the host study and the positive interactions they had with members of the research team was a factor in consultees being likely to agree to participation on behalf of their relative. This view was supported by members of the host study team who described the amended processes for participants who lacked capacity which was intended to reduce the burden of participation.

“I was present during some of the sampling that [husband] had to do for the study, and I know they treated him with care and concern … what more can you ask.” [1015, consultee, control group].

“The type of study that we’re doing, it’s not something that requires a lot of thinking about. Because the [participants] that haven’t got capacity, we don’t do a swab…it’s actually just asking the nurse to take a sample when they go to the toilet.” [SI2, member of host study team].

The consent process for the SWAT was also considered to be acceptable, with no concerns raised by consultees about being approached to take part in the SWAT at the same time as being approached to act as a consultee for the host study. This included being asked to take part in an optional qualitative interview.

“I’ve had no issues with it whatsoever. You know, filling the forms out is easy, talking with you is obviously very easy, so, no issues … It was very easy for me to consent to that.” [1056, consultee, control group].

Based on our previous qualitative research when designing the SWAT [15], the recruitment processes were streamlined as much as possible to avoid over-burdening research teams and consultees. However, in practice, this meant that it was difficult for family members to differentiate between the host study and the SWAT when recalling their experiences as they were viewed as seamlessly integrated, or to recall details about being approached.

“I’m fairly sure that I was approached first of all by the nursing home, by somebody there, who said that a study was going on, and would we be prepared to allow [my sister] to be a part of that study.” [1028, consultee, intervention group].

Consultees were asked to complete the CONCORD scale questionnaire and return it which constituted consent to participate in the SWAT. The host study research team reported that consultees often did not understand they were being asked to complete a separate form as part of the SWAT. They viewed the form as being yet another consent document for the host study that they were completing on behalf of the person lacking capacity.

“It felt like it was part of the consent process really. You know, gathering of information and consent.” [1056, consultee, control group]

“She thought she’d done it because we’d done the consent form, and she had the piece of paper in her hand. Some people were confusing the fact that they’d filled out one form, so why did they need to do another?” [SI2, member of host study team].

There were also challenges with collecting resource use data, as members of the host study team were busy and found it difficult to find time to complete the resource use questionnaire. They also described how, once they had established a standard process for approaching consultees and discussing the host study and SWAT, there was very little variability between the resources needed to approach consultees about the host study and SWAT regardless of whether they were receiving the intervention or control. As a very small research team there was also no variation in the grade or role of staff involved. This meant that they found reporting resource use a repetitive process and struggled to see the value of it.

“That’s something I keep forgetting and have to do like the next day because I always forget to do it at the time. Sometimes it doesn’t go that far because they say no, or you know it comes to the end of the programme and we haven’t actually managed to catch up with them again.” [SI2, member of host study team].

“It’s been at the back of my mind, because every time I was filling it in, I felt like I was just repeating myself on paper every single time I was doing it. So, it just became a bit of an exercise, I kind of forgot about.” [SI2_I2, member of host study team].

### Challenges of measuring decision quality

The difficulties with differentiating the SWAT from the host study influenced the ability to explore the feasibility and acceptability of the CONCORD scale in any great detail. As the CONCORD scale questions were focused on their decision about participation on behalf of the person lacking capacity, and the timing necessarily coincided with being asked to make that decision, consultees often did not understand they were being asked to complete a separate data collection form.

“To me, it felt like it was part of the consent process really. You know, gathering of information, and, obviously, your consent.” [1056, consultee, control group]

The attempt to minimise the perceived burden for consultees may have downplayed the importance of completing the CONCORD scale which in turn impacted on responses to the questions and on return rates.

“… then just as a little extra a sort of questionnaire for you to fill in” [SI2, member of host study team]

This meant that consultees’ recollection of completing the questionnaire was sparse and so it was often difficult to untangle acceptability of the scale from their views about other study processes and documents.

“It was quite lengthy, but I think all the questions in there were relevant, as I recall.” [1056, consultee, control group]

“It was all together, and I wasn’t aware which bit was what.” [1015, consultee, control group]

### Views and experiences of the decision support intervention

The interviews also explored consultees and host study members’ views about the feasibility of the intervention ahead of the main SWAT. This included their experiences of the process of decision making as well as their views about the decision support booklet.

### Making decisions about research

Consultees described how they had deliberated about participation for the person they represented. They reported taking into account the wishes of the person, how they approached making other decisions on their behalf, and weighing up the potential benefits and impact on them.

“Every time I come to make some decision on his behalf, I have to think, and put myself in his shoes, and say what would he want? And that’s what I do for everything I seem to do for him really.” [1015, consultee, control group].

“Unless it was necessary for her health or her needs, I would put myself in her position and say, what would mum think about that, would she agree to that?” [1056, consultee, control group].

Some consultees reported that making a decision on behalf of others can be difficult. Others described feeling confident when making a decision about the host study, although, even when the consultee was experienced in making decisions on behalf of the person they still expressed some uncertainty about whether it was the ‘right thing’ to do.

“I know that this is something that [sister’s name] would, if she’d been approached when she was in her sound mind, certainly would have agreed about. So, I feel confident to say, yes, go ahead with this study, because I really believe that it’s what she would have done. We’ve made so many decisions on her behalf, that you would have thought we would have been quite comfortable, but each decision we have to make, it’s still, have we done the right thing?” [1028, consultee, intervention group]. Consultees were often guided by their own values when making a decision about participation and cited altruistic reasons for making the decision that they should participate. They often used the term ‘we’ or ‘us’ when speaking about research, perceiving it as a joint venture.

“Join a study that would benefit the rest of the world, do you know what I mean? A lot of people are happy to know that they’re just going to help in some small way.” [1015, consultee, control group].

“It was no issue at all. Like I said, if we can help, great.” [1056, consultee, control group]

“We would whole heartedly say yes, go ahead, because it might not help us, but it might help children and grandchildren, etc., as well.” [1028, consultee, intervention group]

### Decision support booklet

Host study staff were very positive about the decision support booklet and saw the value of it being made available to all families coming into hospitals or care homes with relatives who may lack capacity. There were a number of discussions around the randomisation of SWAT participants and members of the host study team questioned whether randomisation was needed or could be done differently such as randomising by site. Unsurprisingly, staff had a clear preference for the decision support booklet over the blank notebook provided to consultees in the control group.

“I do think the decision aid is probably the better one to have, to hand out to next of kins, to give them that extra information. But thinking about notebook, I’m not quite sure if it makes too much of a difference.” [SI2, member of host study team].

However, consultees often had limited recollection about what they had received (decision support booklet or blank notebook), which was echoed by the host study team.

“My memory of reading that information is quite sort of lax at the moment.” [1056, consultee, control group]

“I mean, I liked it. It was colourful, cheerful, clear to read. I just don’t know if people do read these things. In terms of are they being bombarded by too much information, not just about research, but you know at that point in time …” [SI1, member of host study team].

One consultee highlighted how their current situation as a carer for the person they represented impacted on their capacity to read additional information.

“You know, we’ve got a lot of living to do, got people to look after, shopping to do, prescriptions to collect, all sorts of stuff. They haven’t, in the main, got time to sit down and read every minutiae of these booklets and stuff.” [1015, consultee, control group].

“A lot of them were more interested in what we were going to do to their family or friend.” [SI2, member of host study team]

Another consultee who had been randomised to receive the decision support booklet described how it prompted discussion between wider family members they had shared it with, and whilst they could not describe the impact it had on their decision making in any detail, they did report that it had not been a negative experience.

“I think we argued the toss about it, you know. Should we or shouldn’t we, you know. We know a little bit about what is involved, we did ask questions at the time about it and were reassured. It must have been okay, because we went ahead. I can’t remember thinking any sort of adverse things about it.” [1028, consultee, intervention group].

### Delivery of the decision support intervention

Members of the host study team described the process they used for approaching consultees and delivering the intervention. The decision support booklet was introduced to consultees by staff who explained its purpose and suggested consultees could use it to help them to make a decision, although it did not form an active part of the consultation.

“We’ve got our consultee sheets that we group together along with your questionnaire, and we open the envelope to see whether they’ve been given the decision aid or the notebook. We then take it to them and we explain a little bit about our study and then we just say well just to help you make a decision we are going to give you for example a decision aid here which has got a lot of information in there for you to have a little look through, see what you think and that should help you enable to make a decision or we’ve got a notebook here, if you’ve got any questions please write them down so you can bring it back and ask us.” [SI2, member of host study team].

“We’re presenting it together with our study information. So, probably in practice we’re not going through it perhaps in the same way that was originally intended.” [SI, member of host study team].

As it did not form an active part of the consultation, members of the host study team were uncertain whether consultees had used the decision support booklet beyond an initial glance through at the time they were given it.

“I think they mainly just took them with them, or just filled out sheets. Some of them did have a sort of a little skip through when stood there having a chat with them, but nobody really mentioned about the booklet. It was more about the actual study itself.” [SI2, member of host study team].

“To be honest, I don’t really have a lot of experience as to how they’re actually using it when they’re at home, if they’re using it at all.” [SI2, member of host study team]

Members of the host study team did however describe there being a positive response from consultees and described the impact it had on their own research practice of approaching consultees, explaining about their role and supporting them to make a decision.

“The initial reaction [from consultees] was ‘oh, that’s a good thing to have’. Because [being a] consultee is difficult, and no-one prepares you for making that decision on behalf of somebody else. It’s certainly made me think about it.” [SI, member of host study team].

## Discussion

This stage of the SWAT was conducted to explore the feasibility and acceptability of the SWAT intervention and procedures, adding to our previous report of the methodological and ethical considerations we encountered when designing the CONSULT SWAT [23]. The intervention and SWAT procedures were found to be broadly acceptable and feasible, as demonstrated through recruitment and set-up of a host study, without any unintended consequences such as compromising delivery of the host study. It also identified barriers to be addressed ahead of the main SWAT, including the low return rate of CONCORD questionnaires.

However, many of the barriers are commonly encountered when conducting studies involving adults lacking capacity to consent, or when embedding a SWAT, with the CONSULT SWAT occupying a unique intersection between them. For example, the relatively small number of eligible studies from which to select a host study is unsurprising given that adults lacking capacity are frequently excluded from trials [11] and those that do are some of the more challenging trials to conduct [15, 32]. Many of the concerns cited by potential host studies have been encountered by other teams leading co-ordinated SWATs, including a SWAT in paediatric trials [33] where there may be similar ethical and practical issues to those in the CONSULT SWAT. The findings from setting up this SWAT support the recommendations previously made by the PROMETHEUS programme to improve SWAT activity, including the need to increase awareness of the methodological importance of SWAT research with research teams, and that more research is needed to identify the barriers that teams encounter when undertaking a SWAT, and identify strategies and solutions for addressing them [34]. Our qualitative data suggests that the familiarisation process and regular contact between research teams was essential to help understand and address early difficulties in implementing the SWAT in the host study and helped to streamline the processes and achieve a balance between the competing priorities. However, for the CONSULT SWAT, the relatively small number of potential host trials and the low numbers of adults lacking capacity to consent who are recruited (in part due to the burden of decision making for consultees) leads to a circular paradox where there are few trials in which to evaluate the effectiveness of interventions to support recruitment and retention in these trials, or to validate measures such as CONCORD.

This feasibility stage provided the first indication of the likely rates of recruitment of participants for a SWAT involving consultees. As a pre-consent randomisation design was used, 92 family members were randomised to the intervention with 16 completing and returning the CONCORD questionnaire, representing a return rate of 17.4%, and 25% of these were interviewed. The return rates represented 39% (*n* = 16) of those consultees who did agree to the patient/resident participating in the host study (*n* = 41). All of the host study team members who were available for interview were interviewed which provided valuable insights into how and when recruitment challenges occurred. The difference between the numbers randomised and completion of the CONCORD questionnaire (which constituted consent to participate in the SWAT) were primarily due to the well-recognised difficulties associated with approaching family members to act as consultees [7–9], which is the reason for conducting this SWAT. However, family members who declined to act as a consultee, or declined the host study on behalf of the patient/resident, also did not agree to be interviewed and so the reasons they did not agree to participate in the SWAT are unknown. Strategies that are commonly used to take account of missing outcome data from randomised participants in clinical trials, such as using ‘intention to treat’ analysis [35], are not applicable to a SWAT such as this. The pragmatic selection of an observational study rather than a clinical trial to host the feasibility stage may have influenced perceptions about the decision support intervention and affected responses to the CONCORD scale in a number of ways. The host study was perceived to be low risk and not too burdensome for participants which may have led to lower decision burden for consultees, although there was evidence that consultees expressed uncertainty about their decision making including those who were experienced in making other proxy decisions. Previous studies have suggested that whilst there is considerable variation in proxies’ experiences and approach to decision making about research [36], they consider a broad range of factors beyond just the risks and invasiveness of the study itself [7, 10, 37] and nearly all experience some degree of burden when making decisions about research [9].

Whilst the number of participants is low and the groups were of different sizes, with low levels of questionnaire return rates, CONCORD scores were relatively high across both arms. The relatively high CONCORD scores may be reflective of lower levels of decisional burden or decision regret. However, due to combining the SWAT information sheet and questionnaire into one set of documents and presenting it alongside consent documents for the host study the SWAT processes became ‘invisible’. Consultees therefore misunderstood that they were completing a separate questionnaire exploring their experiences, including any uncertainty they felt, rather than confirming their agreement with statements in a consent form. This meant that consultees did not necessarily spend time deliberating about the CONCORD questions as individual items and were less likely to use ‘disagree’ response options—particularly if they were concerned that this would reflect badly on the consent process and the personnel involved.

It is unclear what led to a lack of response to the electronic version of the decision aid and online CONCORD questionnaire, as it also meant that consultees who received the electronic version of the decision aid did not indicate their willingness to be contacted about an interview and so could not be interviewed. Security concerns about clicking on unknown links may have been a factor [38], alongside similar misunderstandings about the purpose or significance of the questionnaire.

The challenges of effectively measuring decision making have been widely reported, including for decisions about research participation [39], not least that simply administering such measures can affect the perception of decision quality or lead to response bias [40]. However, it is possible that the slightly lower CONCORD scores in the intervention arm suggests that the small number who had received the decision aid booklet could have engaged in greater deliberation during the decision-making process and consider issues such as values congruence, rather than making a less deliberation-informed decision that would be considered a lower quality proxy decision [41].

The ability to explore the feasibility of the decision support intervention and CONCORD scale was also affected by the recruitment to the SWAT of only family members who agreed to act as consultees and those who agree to participation in the host study on the person’s behalf. Consultees expressed altruistic views about taking part in research and reported that this view was also shared by the person they represented. This meant that family members with higher levels of uncertainty, who were likely to be less confident in their ability to make a proxy decision or more concerned about making a ‘wrong’ decision, were unlikely to be included in the SWAT. Families where there was conflict between potential consultees, which is not uncommonly encountered in studies involving adults lacking capacity to consent [32], and those of more acutely unwell patients were also not likely to be included. Whilst many studies have previously explored the attitudes and behaviour of research staff that can influence recruitment and retention in trials [42], this has yet to be explored in the context of recruiting adults lacking capacity. Although research staff are known to be apprehensive when recruiting participants with impaired capacity due to the accompanying ethical and legal issues which they can view as being a ‘black box of horrendousness’ [15].

The decision support intervention was considered acceptable to both consultees and host study staff. Consultees had limited recall of having received either the decision aid or notebook which impacted on the ability to explore any effect of the intervention on the quality of the decision, defined as informed values‐based choice congruence [43], although assessing effectiveness is an objective of the main SWAT rather than this feasibility stage. Encouragingly, there did not appear to be any adverse consequences, such as impacting their confidence in decision‐making or reports of decision regret or emotional distress [43]. It appeared that the intervention was not necessarily delivered as planned, with less interaction with the decision aid booklet during the consultation than intended. However, this may reflect its likely use in practice with varying levels of interactivity depending on the decision-making context. Members of the host study team did report that the intervention had positively impacted on their own research practice, which was one of the aims of the intervention specified in the logic model for the intervention [20].

One of the objectives of the feasibility stage was to explore the costs of the intervention in terms of its impact on the research delivery system (e.g. consultation length) rather than its cost-effectiveness since societal preferences for improved decision quality are unknown [20]. The costs associated with printing and posting hard copies of the DA and questionnaires were small, but it proved challenging to elicit any further detailed information about the resources used to deliver it, including the consultee’s time. The slightly higher resource use in the intervention group was primarily associated with one outlier and was due to issues unrelated to the intervention. There was no indication from the qualitative data that the intervention group was any more resource intensive than the control group but that may be due to it not being delivered as intended, with consultations being relatively brief. The focus on efficiency likely reflects the competing pressure to recruit to the host study, and to deliver studies to time and to target as outlined in the UK’s Future of Clinical Research Delivery plan [44].

### Recommended changes to CONSULT SWAT processes

To help address these issues, the SWAT processes and guidance documents have been updated to reflect a better balance between minimising the burden for all those involved and improving data quality. This includes two key areas:

- The training provided to host study team about the SWAT and the supporting manual were updated with additional suggested phrases for emphasising to consultees that (1) the CONCORD questionnaire is a separate to the consent documents, (2) it is important to complete the questionnaire as it helps us learn more about the experiences of families involved in decisions about research and (3) responses to the questionnaire do not reflect badly on the consent process or those involved.
- Members of the host study team offered consultees a stamped addressed envelope to return the CONCORD questionnaire to help (1) facilitate return of completed questionnaires, (2) distinguish between the questionnaire and consent documents and (3) enable consultees to indicate if they do not agree with the statements as their responses would not be immediately visible to the host study team member.

Additional suggestions to consider prior to the main SWAT include a ‘streamlining’ of SWAT processes such as a mechanism for host study teams to track electronic completion of the CONCORD scale and considering alternative methods for host trial to record screening and randomisation of SWAT participants and linking with the host study participant via a single spreadsheet rather than across a number of logs. Other recommendations included providing alternative electronic access to the CONCORD questionnaire via a QR code and simplifying the process for collecting resource use data.

### Strengths and limitations

In common with the challenges encountered in many studies when approaching family members to act as a consultee, a relatively low number of family members who had been randomised in this stage of the SWAT provided consent to participate, as confirmed by the low return rate of the CONCORD questionnaire. Consultees who did agree to participate had known the person they represented in a broad range of familial and spousal relationships, which represents those often encountered in practice. However, as only those who agreed to act as consultee and agreed to person’s participation in the host study were included in the SWAT, and they were small in number, their views may not be representative of other family members who declined and who may have different experiences of decision-making or less altruistic views about research. Although recruitment logs were used to identify which family members had been approached and randomised to the SWAT in relation to potential participants, the exact number who either did not respond, declined to act as consultee or declined the study on the person’s behalf are difficult to ascertain due to the complexities of not necessarily being able to track and follow family members who may potentially act as a consultee, unlike the ability to track patients/participants themselves. Family members of patients with more acute health and care needs were also less likely to be approached to take part, and some of those who did agree to participate in an interview for the SWAT later withdrew due to unexpected or more pressing issues with the person’s care. It is possible that all these groups of family members may have benefited from decision support to differing degrees. The host study was a low-risk observational study rather than an RCT which may have affected family members’ decisions about participation.

A strength of the study was that SWAT participants were randomised to receive the decision support booklet or a notebook in the control arm and were allocated as planned. However, fewer consultees in the intervention group completed and returned the questionnaire, and hence they contributed less CONCORD data. As consent to be contacted for an interview was obtained via the same form as the CONCORD questionnaire, we were not able to interview consultees who did not return the questionnaire. Only one consultee who had received the intervention was interviewed. The interviews and co-analysis of the data were conducted by an experienced qualitative researcher who was not involved in developing the intervention or designing the SWAT, which brought an additional and impartial perspective to the qualitative findings.

Integrating the SWAT into the host study and streamlining the processes in order to minimise the time and burden for those involved meant that it was rendered invisible, and the small number of consultees interviewed found it hard to recall details about being approached.

### Implications for future SWATs

The findings from conducting this feasibility stage of the SWAT will be useful to those conducting similar SWATs in the future, as well as those evaluating similar interventions. We have previously reported the methodological considerations we encountered when designing the CONSULT SWAT [23], and Table 4 provides an updated summary of the main considerations for future SWATs, together with our proposed solutions to help address some of these issues.

Whilst not an intended focus of the SWAT interviews, family members shared their views about being involved in the host study which could be valuable feedback for hosts studies and seen as a potential benefit of embedding a SWAT.

**Table 4:** Summary of the main methodological considerations for SWATs in trials involving adults lacking capacity and proposed solutions. (Additional suggestions from CONSULT SWAT feasibility stage in italics)

Methodological area	Description of the issue	Recommendations
Maintaining the integrity of the host trial	Impact on recruitment and retention rates from interventions aimed at improving proxy decision-making is unclear	• Undertake assessment of host trial context to ensure suitability for the SWAT and anticipate issues with embedding the intervention and/or SWAT • Adopt an exploratory approach to obtaining and analysing recruitment and retention data in host trials • Record and report factors affecting intervention effectiveness and/or implementation, and impact on the host trial • *An initial mixed methods feasibility stage using a rapid qualitative evaluation approach may be useful to help provide timely insights into challenges that can be addressed prior to the main SWAT*
Identifying a suitable outcome measure	Less methodological research in trials involving adults lacking capacity, therefore less known about appropriate outcomes and measurement instruments	• Factor in the need for preliminary work to establish relevant outcomes and outcome measurement instruments (including timing) • Consider whether work is needed to develop or adapt (and validate) outcome measurement instruments prior to SWAT • *Consider the small sample sizes in studies involving adults lacking capacity, which may require a larger number of host trials when validating measures and assessing outcomes than for SWATs in other populations* • *Explore the acceptability and feasibility of using alternative data collection methods, such as electronic tools*
Unpredictability of sample sizes	SWAT sample size is dependent on the host trial which may be more heterogenous and have greater uncertainty than for SWATs in other populations	• Work with the host trial team to assess the likely proportion of participants who will lack capacity (as a whole and by site) and the proportion expected to have a personal consultee/legal representative • Encourage reporting of capacity status and use of consultees/legal representatives in relevant trials to inform future SWATs
Challenges in consent and data collection	SWAT participant is not generally a participant in the host trial and so does not usually provide their own consent or data for the host trial	• Incorporate flexibility into the design of the SWAT to enable alignment with host trial processes and so minimise burden for trials and SWAT participants • *Ensure that the SWAT maintains a distinct identity to that of the host study to avoid participants misunderstanding or missing the significance of documents that form part of the SWAT rather than the consent process for the host trial* • *Consider the impact of family members managing competing demands due to the health and care needs of the person they represent* • *Include suggested phrases in the training for site staff about how best to introduce the SWAT and host trial in different contexts* • *To minimise recruitment bias, encourage site staff to include family members in the SWAT regardless of their decision about whether to agree to participation in the host trial or not* • *Identify opportunities for flagging potential SWAT participants, for example adding an alert in the host study database to flag when a host study participant is assessed as lacking capacity to consent* • *Questionnaire response rates may be lower than anticipated, therefore consider pre-emptively implementing strategies to maximise return rates*
Uncertainties about the resources needed to deliver the intervention	Trials involving adults lacking capacity are more resource-intensive and so determining the cost of delivering recruitment/retention interventions and conducting SWATs is particularly important in these trials	• Explore how best to collect resource use data from proxies as non-participants in the host trial • Costs attributed to delivering the intervention and SWAT need to be disentangled from the costs of delivering the host trial • Additional work is needed to determine the most appropriate perspective for economic evaluations in these SWATs • *Work with the host trial team to identify the least burdensome method for collecting resource use data*
Selecting an appropriate randomisation strategy	Unlike in ‘traditional’ SWATs, decisions about randomisation need to take account that proxies are not participants in the host trial, in addition to common questions about appropriate level of randomisation	• Randomisation strategies (and processes) in SWATs involving adults lacking capacity may need to be flexible to align with host trials • Process evaluation may be needed to capture any contamination, differential recruitment, and/or adaptations which may undermine intervention fidelity
Considerations for analysis	Meta-analyses will need to consider the heterogeneity between trials and populations, and take account of the differences in SWAT implementation between host trials	• Meta-analysis will need to consider the impact of different levels of randomisation, delivery of the intervention, timing of outcome measurement etc • Judgements will be needed about the appropriateness of including all host trials in a meta-analysis, and the need for sub-group analyses • *Consider how trials with very small numbers of adults lacking capacity (and therefore low numbers of potential SWAT participants) will be handled during analysis*

Table adapted from: Shepherd, V., Wood, F., Gillies, K. et al. Recruitment interventions for trials involving adults lacking capacity to consent: methodological and ethical considerations for designing Studies Within a Trial (SWATs). Trials 23, 756 (2022). 10.1186/s13063-022-06705-y

## Conclusion

This study has demonstrated that it is feasible to conduct the CONSULT SWAT, although with changes needed to study processes prior to continuing to the main SWAT stage. In addition to informing the main SWAT, the findings have been used to refine the recommendations for designing and conducting future SWATs in this population and others where additional ethical and methodological considerations arise.

The circular paradox of there being a small number of potential host trials and participants who lack capacity to consent can limit opportunities for trials methodology research in this area, including validating outcome measures such as CONCORD. Unless action is taken to address this, it will remain a barrier to the equitable development of evidence-based interventions for studies involving this population. Future strategies could include the NIHR and other funders incentivising trial teams to embed SWATs in studies involving under-served groups where there are additional ethical and methodological challenges, without incurring a risk to the continuation of the host trial.

Recruitment of host trials for CONSULT SWAT is underway, with a particular focus on evaluating the decision support intervention in a broad range of populations, settings and types of trials. We encourage replication of the SWAT in other trials involving adults with impaired capacity to consent. Due to the evolving use of different methods for contacting and consulting with family members, future research could include exploring the provision of the decision aid and CONCORD scale in alternative formats.

## Supplementary Information

Supplementary Material 1.

Supplementary Material 2.

## Data Availability

The dataset that will be generated in this study will be available through submission of a data request to the Centre for Trials Research at https://www.cardiff.ac.uk/centre-for-trials-research/about-us/data-requests

## References

[1] Health Research Authority. Health research authority: mental capacity act. Health research authority. https://www.hra.nhs.uk/planning-and-improving-research/policies-standards-legislation/mental-capacity-act/. Accessed 16 Jul 2021.

[2] The Medicines for Human Use (Clinical Trials) Regulations. 2004. SI No.1031 https://www.legislation.gov.uk/. 15812991

[3] HMSO, London. Mental capacity act 2005. 2005.

[4] Adults with Incapacity (Scotland) Act. 2000. https://www.legislation.gov.uk/.

[5] Mental Capacity Act (Northern Ireland). 2016. https://www.legislation.gov.uk/. 10.1192/pb.bp.117.056945 PMC5709686 29234514

[6] Shepherd V. Research involving adults lacking capacity to consent: the impact of research regulation on “evidence biased” medicine. BMC Med Ethics. 2016;17:8. 27609355 10.1186/s12910-016-0138-9 PMC5016956

[7] Shepherd V, Hood K, Sheehan M, Griffith R, Wood F. ‘It’s a tough decision’: a qualitative study of proxy decision-making for research involving adults who lack capacity to consent in UK. Age Ageing. 2019. 10.1093/ageing/afz115. 31595291 10.1093/ageing/afz115

[8] Iverson E, Celious A, Kennedy CR, Shehane E, Eastman A, Warren V, et al. Real-time perspectives of surrogate decision-makers regarding critical illness research: findings of focus group participants. Chest. 2012;142:1433–9. 22677349 10.1378/chest.11-3199 PMC3515024

[9] Sugarman J, Cain C, Wallace R, Welsh-Bohmer KA. How proxies make decisions about research for patients with Alzheimer’s disease. J Am Geriatr Soc. 2001;49:1110–9. 11555076 10.1046/j.1532-5415.2001.49218.x

[10] Mason S, Barrow H, Phillips A, Eddison G, Nelson A, Cullum N, et al. Brief report on the experience of using proxy consent for incapacitated adults. J Med Ethics. 2006;32:61–2. 16373526 10.1136/jme.2005.012302 PMC2563273

[11] Shepherd V, Wood F, Griffith R, Sheehan M, Hood K. Protection by exclusion? The (lack of) inclusion of adults who lack capacity to consent to research in clinical trials in the UK. Trials. 2019. 10.1186/s13063-019-3603-1. 31382999 10.1186/s13063-019-3603-1 PMC6683336

[12] Witham MD, Anderson E, Carroll C, Dark PM, Down K, Hall AS, et al. Developing a roadmap to improve trial delivery for under-served groups: results from a UK multi-stakeholder process. Trials. 2020;21:694. 32738919 10.1186/s13063-020-04613-7 PMC7395975

[13] Nuffield Council on Bioethics. The future of ageing: ethical considerations for research and innovation. Nuffield Council on Bioethics. 2023. https://www.nuffieldbioethics.org/publication/the-future-of-ageing-ethical-considerations-for-research-and-innovation. Accessed 14 Aug 2025

[14] World Health Organisation. Guidance for best practices for clinical trials. World Health Organisation (WHO). 2024. https://www.who.int/publications/i/item/9789240097711. Accessed 14 Aug 2025.

[15] Shepherd V, Hood K, Wood F. Unpacking the ‘Black Box of Horrendousness’: a qualitative exploration of the barriers and facilitators to conducting trials involving adults lacking capacity to consent. Trials. 2022. 10.1186/s13063-022-06422-6. 10.1186/s13063-022-06422-6 PMC9167903 35668460

[16] Gillies K, Campbell MK. Development and evaluation of decision aids for people considering taking part in a clinical trial: a conceptual framework. Trials. 2019;20:401. 31277693 10.1186/s13063-019-3489-y PMC6612082

[17] Hudek N, Carroll K, Semchisen S, Vanderhout S, Pressau J, Grimshaw J, et al. Describing the content of trial recruitment interventions using the TIDieR reporting checklist: a systematic methodology review | BMC Medical Research Methodology | Full Text. Trials. 2024;24:85. 10.1186/s12874-024-02195-5 PMC11000410 38589803

[18] Bower P, Brueton V, Gamble C, Treweek S, Smith CT, Young B, et al. Interventions to improve recruitment and retention in clinical trials: a survey and workshop to assess current practice and future priorities. Trials. 2014;15:399. 25322807 10.1186/1745-6215-15-399 PMC4210542

[19] CONSULT. Cardiff University. https://www.cardiff.ac.uk/centre-for-trials-research/research/studies-and-trials/view/consult. Accessed 2 Sep 2024.

[20] Shepherd V, Wood F, Griffith R, Sheehan M, Hood K. Development of a decision support intervention for family members of adults who lack capacity to consent to trials. BMC Med Inform Decis Mak. 2021;21:30. 33509169 10.1186/s12911-021-01390-4 PMC7842028

[21] Shepherd V, Wood F, Gillies K, Martin A, O’Connell A, Hood K. Feasibility, effectiveness and costs of a decision support intervention for consultees and legal representatives of adults lacking capacity to consent (CONSULT): protocol for a randomised study within a trial. Trials. 2022;23:957. 36434661 10.1186/s13063-022-06887-5 PMC9701035

[22] Treweek S, Bevan S, Bower P, Campbell M, Christie J, Clarke M, et al. Trial forge guidance 1: what is a study within a trial (SWAT)? Trials. 2018;19:139. 29475444 10.1186/s13063-018-2535-5 PMC5824570

[23] Shepherd V, Wood F, Gillies K, O’Connell A, Martin A, Hood K. Recruitment interventions for trials involving adults lacking capacity to consent: methodological and ethical considerations for designing studies within a trial (SWATs). Trials. 2022;23:756. 36068637 10.1186/s13063-022-06705-y PMC9450319

[24] The Northern Ireland Network for Trials Methodology Research, Queen’s University Belfast. SWAT repository store. http://www.qub.ac.uk/sites/TheNorthernIrelandNetworkforTrialsMethodologyResearch/SWATSWARInformation/Repositories/SWATStore/. Accessed 11 Oct 2019.

[25] Arundel CE, Clark LK, Parker A, Beard D, Coleman E, Cooper C, et al. Trial forge guidance 4: a guideline for reporting the results of randomised studies within a trial (SWATs). Trials. 2024;25:183. 38475795 10.1186/s13063-024-08004-0 PMC10935912

[26] Eldridge SM, Chan CL, Campbell MJ, Bond CM, Hopewell S, Thabane L, et al. Consort 2010 statement: extension to randomised pilot and feasibility trials. Pilot Feasibility Stud. 2016;2:64. 27965879 10.1186/s40814-016-0105-8 PMC5154046

[27] Shepherd V, Hood K, Gillies K, Wood F. Development of a measure to assess the quality of proxy decisions about research participation on behalf of adults lacking capacity to consent: the combined scale for proxy informed consent decisions (CONCORD scale). Trials. 2022;23:843. 36195929 10.1186/s13063-022-06787-8 PMC9531498

[28] Vindrola-Padros C. Doing rapid qualitative research. London: SAGE Publications Ltd; 2021.

[29] Braun V, Clarke V. Reflecting on reflexive thematic analysis. Qual Res Sport Exerc Health. 2019;11:589–97.

[30] Vindrola-Padros C, Chisnall G, Polanco N, Vera San Juan N. Iterative cycles in qualitative research: introducing the RREAL sheet as an innovative process. 2022.

[31] DHSC, NHS. Reset programme bulletin. 2022.

[32] Shepherd V. An under-represented and underserved population in trials: methodological, structural, and systemic barriers to the inclusion of adults lacking capacity to consent. Trials. 2020;21:445. 32471488 10.1186/s13063-020-04406-y PMC7257506

[33] Martin-Kerry J, Parker A, Bower P, Watt I, Treweek S, Torgerson D, et al. Swatted away: the challenging experience of setting up a programme of SWATs in paediatric trials. Trials. 2019;20:141. 30782209 10.1186/s13063-019-3236-4 PMC6381684

[34] Parker A, Arundel C, Clark L, Coleman E, Doherty L, Hewitt CE, et al. Undertaking studies within a trial to evaluate recruitment and retention strategies for randomised controlled trials: lessons learnt from the PROMETHEUS research programme. Health Technol Assess. 2024;28:1–114. 38327177 10.3310/HTQW3107 PMC11017159

[35] White IR, Horton NJ, Carpenter J, Statistics R, in M and S, Pocock SJ. Strategy for intention to treat analysis in randomised trials with missing outcome data. BMJ. 2011;342: d40. 21300711 10.1136/bmj.d40 PMC3230114

[36] Hosie A, Kochovska S, Ries N, Gilmore I, Parker D, Sinclair C, et al. Older persons’ and their caregivers’ perspectives and experiences of research participation with impaired decision-making capacity: a scoping review. Gerontologist. 2022;62:e112–22. 32866239 10.1093/geront/gnaa118

[37] Bogaerts JMK, Warmerdam LA, Achterberg WP, Gussekloo J, Poortvliet RKE. Proxy decision-making for clinical research in nursing home residents with dementia: a qualitative analysis. J Am Med Dir Assoc. 2023;24:541-547.e2. 36924797 10.1016/j.jamda.2023.02.017

[38] Laukka E, Lakoma S, Harjumaa M, Hiltunen S, Härkönen H, Jansson M, et al. Older adults’ preferences in the utilization of digital health and social services: a qualitative analysis of responses to open-ended questions. BMC Health Serv Res. 2024;24:1184. 39367429 10.1186/s12913-024-11564-1 PMC11451244

[39] Gillies K, Elwyn G, Cook J. Making a decision about trial participation: the feasibility of measuring deliberation during the informed consent process for clinical trials. Trials. 2014;15:307. 25073967 10.1186/1745-6215-15-307 PMC4131044

[40] Norman GR, Cairney J. Health measurement scales: a practical guide to their development and use. 5th Edition. Oxford: Oxford University Press; 2015.

[41] Shepherd V. (Re)Conceptualising ‘good’ proxy decision-making for research: the implications for proxy consent decision quality. BMC Med Ethics. 2022;23:75. 35850682 10.1186/s12910-022-00809-5 PMC9294776

[42] Coffey T, Duncan EM, Morgan H, Lawrie L, Gillies K. Behavioural approaches to recruitment and retention in clinical trials: a systematic mapping review. BMJ Open. 2022;12: e054854. 35264354 10.1136/bmjopen-2021-054854 PMC8915327

[43] Stacey D, Lewis KB, Smith M, Carley M, Volk R, Douglas EE, et al. Decision aids for people facing health treatment or screening decisions. Cochrane Database Syst Rev. 2024. 10.1002/14651858.CD001431.pub6. 10.1002/14651858.CD001431.pub6 PMC10823577 38284415

[44] The future of clinical research delivery: 2022 to 2025 implementation plan. Department of Health and Social Care. 2022. https://www.gov.uk/government/publications/the-future-of-uk-clinical-research-delivery-2022-to-2025-implementation-plan. Accessed 14 Aug 2025

