# Supplementary material for: Feasibility of a Study Within a Trial to evaluate a decision support intervention for families deciding about research on behalf of adults lacking capacity to consent (CONSULT SWAT)
Source: Trials. 2025 Aug 27;26:313. doi: 10.1186/s13063-025-09021-3 (PMC12382041; doi:10.1186/s13063-025-09021-3)
Supplement: Supplementary file 1 — Supplementary Material 1. [file 13063_2025_9021_MOESM1_ESM.doc]

**CONSULT Interview Topic Guide – Family Members**

**Introduction**

**Experiences of CONSULT**

- Please could you tell me a bit about what led up to you taking part in the CONSULT Study?

>Prompt:

- Were you approached by a member of your relatives’ care team?
- How did that come about?
- How much did you know about research before taking part in this study?
- How much did you know about your involvement in making a decision about research on their behalf before taking part in this study?

**Acceptability of study information and consent process**

- What information were you given about the CONSULT Study?
- What did you think about the information you were given?
- Did you have any questions about the study at the time? Were you able to have these answered fully?
- How did you feel about taking part in the CONSULT Study?
- Are there any ways in which the study information could be improved? Is there anything you wish you had known about the research?
- Can you think of any ways in which the study consent process could be improved?

**Decision support booklet**

I’m now going to ask you some questions about <host trial name>

- Did you receive a booklet called ‘Making decisions about research’ as part of the information about <host trial name>?
- Did you go through the booklet? How did you use the booklet?
- How long did it take you to go through the booklet?
- What did you think about the booklet?
- Did you think it was helpful or not helpful when you were thinking about whether your relative should take part in the <name of host trial>?
- Were there particular sections that you found helpful or not helpful?
- What kinds of things might make improve the booklet? Why?
- What kinds of things might make it easier for the booklet to be used? Why?

**Making a decision**

- How did you feel about making a decision about whether your relative would take part in <name of host trial> when you were first approached?
- How did you go about making a decision? What sort of information did you use? Did you speak to anyone else?
- Did the booklet affect how you made a decision? How?
- How do you feel now having made that decision?

**Data collection**

- Do you remember being given a questionnaire to complete as part of the CONSULT Study?
- What did you think about this? Did you see this as part of the consent process for your relative to take part in the host trial?
- How easy or difficult was it to complete the questionnaire you were given?
- Are there any questions that were difficult to answer? Why?
- Could any improvements be made to the paperwork or questionnaire?

**Overall acceptability**

- What was it like being part of the CONSULT Study?
- Were there any problems with the CONSULT Study that you haven’t already mentioned?
- Can you think of any other ways that the CONSULT Study could be improved?

**End of interview**

- We’ve covered all of the questions I wanted to talk about today, is there anything we haven’t mentioned that you would like to say?
- Thank you very much for taking the time to talk to me today

**Debrief**
