# Supplementary material for: Feasibility of a Study Within a Trial to evaluate a decision support intervention for families deciding about research on behalf of adults lacking capacity to consent (CONSULT SWAT)
Source: Trials. 2025 Aug 27;26:313. doi: 10.1186/s13063-025-09021-3 (PMC12382041; doi:10.1186/s13063-025-09021-3)
Supplement: Supplementary file 2 — Supplementary Material 2. [file 13063_2025_9021_MOESM2_ESM.docx]

**RREAL Sheet Consultee**

| **Information** | **Summary** |
| --- | --- |
| Experience of CONSULT |  |
| Acceptability of study information and consent process |  |
| Decision support booklet |  |
| Making a decision |  |
| Data collection |  |
| Overall acceptability |  |
| Other information |  |
